# Legal Notes for Institutional Workers

**Published:** 1917-07-14

**Authors:** 


### LEGAL NOTES FOR INSTITUTIONAL WORKERS.

### Who is Responsible for the Insurance Payments for Nurses?

The Court of King's Bench in Ireland have lately decided a point of some interest under the National Insurance Act, *Irish Insurance Commissioners* v. *Enniskillen Poor Guardians*. The medical officer of the defendants required a nurse for the infirmary, and he communicated with a Miss Reid, the proprietor of a nurses' home in Derry, who sent him a lady on the terms and conditions of a printed prospectus issued by her, under which in substance the nurse was to carry out her duties under the control of the doctor and matron, but the salary was collected by the home and 5s. 6d. weekly deducted in respect of its maintenance and upkeep, and when the nurse was unemployed she had still to pay this sum weekly to the institution. The Guardians, contending that they were not the employers of the nurse, refused to pay the insurance contribution, and were summoned for default before the justices. The magistrates by a majority held as a fact that the Guardians were not the employers, but, at the request of the plaintiffs, stated a case for the opinion of the King's Bench Division. The contention of the defendants was that the proprietor of the nurses' home was the employer of the nurse, and that, the justices having so found, there was an end of the case. The Insurance Commissioners argued that there was no evidence to sustain the decision that the nurse was employed by the nursing institution. The Court unanimously decided that there was evidence of fact both ways, and that it was peculiarly the function of the justices to decide such a question, and therefore they upheld the decision of the Court below.

